# A Wireless and Wearable Multimodal Sensor to Non-Invasively Monitor Transabdominal Placental Oxygen Saturation and Maternal Physiological Signals

**DOI:** 10.3390/bios14100481

**Published:** 2024-10-07

**Authors:** Thien Nguyen, Soongho Park, Asma Sodager, Jinho Park, Dahiana M. Gallo, Guoyang Luo, Roberto Romero, Amir Gandjbakhche

**Affiliations:** 1 *Eunice Kennedy Shriver* National Institute of Child Health and Human Development, National Institutes of Health, 49 Convent Drive, Bethesda, MD 20892, USA; thien.nguyen4@nih.gov (T.N.); soongho.park@nih.gov (S.P.); jinho.park@nih.gov (J.P.); 2 Laboratory of Vascular Thrombosis and Inflammation, National Heart, Lung, and Blood Institute, National Institutes of Health, 49 Convent Dr., Bethesda, MD 20814, USA; 3 Department of Obstetrics and Gynecology, Wayne State University School of Medicine, 3990 John R. Street, Detroit, MI 48201, USA; dahiana.gallo@sluhn.org; 4 Division of Maternal-Fetal Medicine, Department of Obstetrics & Gynecology, St. Luke’s University Health Network, 701 Ostrum Street, Suite 303, Bethlehem, PA 18015, USA; 5 Obstetrics & Gynecology at the University of Virginia School of Medicine, 1340 Jefferson Park Ave, Charlottesville, VA 22903, USA; guoyang.luo@inova.org; 6 Department of Obstetrics & Gynecology, Division of Maternal Fetal Medicine, Fairfax Hospital, 3300 Gallows Rd, Falls Church, VA 22042, USA; 7 Pregnancy Research Branch, Division of Obstetrics and Maternal-Fetal Medicine, Division of Intramural Research, *Eunice Kennedy Shriver* National Institute of Child Health and Human Development, National Institutes of Health, U.S. Department of Health and Human Services, Bethesda, MD 20892, USA; romeror@mail.nih.gov; 8 Department of Obstetrics and Gynecology, University of Michigan, Ann Arbor, MI 48109, USA; 9 Department of Epidemiology and Biostatistics, Michigan State University, East Lansing, MI 48824, USA

**Keywords:** near-infrared spectroscopy, intrauterine hypoxia, transabdominal placental oxygenation

## Abstract

Poor placental development and placental defects can lead to adverse pregnancy outcomes such as pre-eclampsia, fetal growth restriction, and stillbirth. This study introduces two sensors, which use a near-infrared spectroscopy (NIRS) technique to measure placental oxygen saturation transabdominally. The first one, an NIRS sensor, is a wearable device consisting of multiple NIRS channels. The second one, a Multimodal sensor, which is an upgraded version of the NIRS sensor, is a wireless and wearable device, integrating a motion sensor and multiple NIRS channels. A pilot clinical study was conducted to assess the feasibility of the two sensors in measuring transabdominal placental oxygenation in 36 pregnant women (n = 12 for the NIRS sensor and n = 24 for the Multimodal sensor). Among these subjects, 4 participants had an uncomplicated pregnancy, and 32 patients had either maternal pre-existing conditions/complications, neonatal complications, and/or placental pathologic abnormalities. The study results indicate that the patients with maternal complicated conditions (69.5 ± 5.4%), placental pathologic abnormalities (69.4 ± 4.9%), and neonatal complications (68.0 ± 5.1%) had statistically significantly lower transabdominal placental oxygenation levels than those with an uncomplicated pregnancy (76.0 ± 4.4%) (*F* (3,104) = 6.6, *p* = 0.0004). Additionally, this study shows the capability of the Multimodal sensor in detecting the maternal heart rate and respiratory rate, fetal movements, and uterine contractions. These findings demonstrate the feasibility of the two sensors in the real-time continuous monitoring of transabdominal placental oxygenation to detect at-risk pregnancies and guide timely clinical interventions, thereby improving pregnancy outcomes.

## 1. Introduction

Intrauterine hypoxia, a critical condition in pregnancy that is characterized by a deficiency in oxygen supply to the fetus, poses significant risks if undetected, potentially leading to adverse pregnancy outcomes [1]. If left untreated, it can result in the redistribution of blood towards the fetal brain, heart, and upper extremities, which can eventually lead to a decline in cardiac function [2]. In addition, it may cause an increased risk of hypertension, cardiovascular disease, abnormal central nervous system development, and cerebral palsy in the fetus [1,3,4]. Habek reported that the occurrence of sudden infant death syndrome is preconditioned by intrauterine hypoxia [3]. Intrauterine hypoxia can occur in two forms, namely pre-placental and placental hypoxia [1,2]. Pre-placental hypoxia happens due to a low blood oxygen saturation level in the mother [1,2]. On the other hand, placental hypoxia can be caused by impaired uteroplacental circulation [1]. The timely and accurate monitoring of maternal physiological signals and placental oxygenation levels allows for the early detection of intrauterine hypoxia, and, as a result, enables timely interventions to improve pregnancy outcomes. 

Various technologies, including Doppler ultrasound, functional Magnetic Resonance Imaging (fMRI), and near-infrared spectroscopy (NIRS), have been employed to assess placental dysfunction and identify the pregnancies at risk. While Doppler ultrasound is commonly used in clinical settings, it cannot measure placental oxygenation and has limitations such as technique dependence, susceptibility to motion artifact, and a lack of wearability [5]. On the other hand, though fMRI has emerged as a promising non-invasive tool for exploring placental perfusion and oxygenation, the widespread clinical adoption of fMRI is impeded by factors such as high costs, limited availability, and potential safety concerns, particularly in early pregnancy. Recently, NIRS, a non-invasive optical imaging technology, has been explored in assessing real-time tissue oxygenation. A key advantage of NIRS lies in its ability to directly measure placental tissue oxygenation.

Razem et al. assessed transabdominal placental oxygenation in active labor using cardiotocography (CTG) and NIRS. They reported that the placental deoxygenations detected by NIRS were related to fetal/neonatal acidosis, which showed that NIRS is more reliable than CTG in identifying this condition [6]. In addition, Wang et al. evaluated placental oxygenation using NIRS in pregnant women with an anterior placenta during the third trimester [7]. They found no difference in baseline placental oxygenation between women with a normal pregnancy and those with an adverse pregnancy outcome, nor any difference between normal and malperfused placentas. In another study using NIRS, Hasegawa et al. found that, compared to an appropriate-for-gestational-age group, the placental oxygenation level immediately before delivery was significantly higher in a small-for-gestational-age (SGA) group with severe pre-eclampsia and placental abnormalities, but significantly lower in the SGA group with umbilical cord abnormalities [8]. An advantage of NIRS is that it can be made miniature, wearable, and wireless. However, the NIRS systems used in the previously mentioned studies were cumbersome and/or bulky. 

Wearable technology has shown great promise in measuring physiological signals continuously, including temperature, respiration, and heart rate, at a variety of locations, including the head, eyes, mouth, chest, arm, wrist, finger, waist, leg, and feet [9,10,11]. In this project, we have developed two wearable, NIRS-based sensors to monitor placental tissue oxygen saturation transabdominally. The first one, the NIRS sensor, which consists of multiple NIRS channels, was used to obtain the tissue oxygenation levels at the placenta of 12 singleton pregnant women [12]. The measurement from the NIRS sensor suggests a close relationship between placental oxygenation level and pregnancy complications/placental abnormal pathologies [12]. This result was published in our previous manuscript [12]. The second one, the Multimodal sensor, which is an upgraded version of the NIRS sensor, is capable of simultaneously measuring transabdominal placental oxygenation, maternal physiological signals, and fetal movement. The Multimodal sensor was utilized to collect data from 24 pregnant women. For the statistical analysis on placental oxygenation, the data collected from both sensors (*n* = 36) were combined. However, for the other parameters, which could only be detected by the Multimodal sensor, the data were analyzed separately *(n* = 24). This study hypothesizes that maternal pre-existing conditions/complications, placental pathologic abnormalities, as well as neonatal complications negatively affect placental oxygenation levels. We believe that the innovation of NIRS technology opens avenues for non-invasive continuous monitoring and personalized care in the realm of obstetrics, marking a significant step toward improving maternal, fetal, and neonatal outcomes. 

## 2. Materials and Methods

### 2.1. Multimodal Sensor

**Figure 1:**
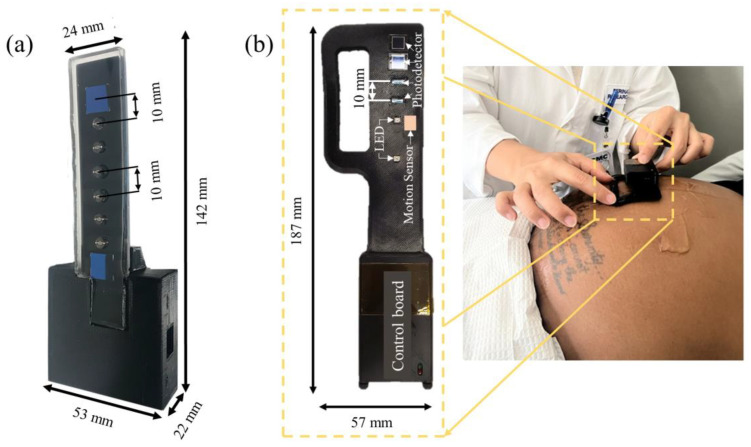
(**a**) The NIRS sensor, consisting of multiple NIRS channels to measure tissue oxygenation; (**b**) the Multimodal sensor, consisting of a motion sensor and multiple NIRS channels to measure tissue oxygenation, maternal physiological signals, and fetal movement. LED: light-emitting diode.

This study introduces two sensors, which have both been developed based on the NIRS technique. The first one, the NIRS sensor, consists of six dual-wavelength, light-emitting-diodes (LEDs) (760 nm and 840 nm) (L760/840-05A, Ushio Semiconductors, Tokyo, Japan) and two photodiodes (S12915-66R, Hamamatsu photonics, Shizuoka, Japan) (Figure 1a). The data acquisition rate of the NIRS sensor is 0.5 Hz. Detailed specifications of the NIRS sensor can be found in our previous publication [12]. The second one, the Multimodal sensor, weighs approximately 61.2 g, with a thickness of 15 mm, a width of 57 mm, and a length of 187 mm (Figure 1b). It consists of a control board covered in a plastic enclosure and a probe covered with flexible polydimethylsiloxane (PDMS). The control board contains a microcontroller (AFE4950, Texas Instruments, Washington, DC, USA) to control the probe, a Bluetooth module (MDBT42Q, Raytac Corporation, New Taipei, Taiwan) to receive and transfer data, and a battery (C1854 Li-Polymer, Shenzhen Pkcell Battery Co., Ltd., Shenzhen, China) as a power supply. The sensor has a battery life of 72 h. The probe comprises two LEDs (SMT735/810/850, Ushio Semiconductors, Tokyo, Japan), 4 photodetectors (PDs) (S9345, Hamamatsu photonics, Shizuoka, Japan), and an embedded accelerometer (MMA8652FC, NXP Semiconductor, Eindhoven, Netherlands) as a motion sensor. The LEDs emit light at 735 nm, 810 nm, and 850 nm, with an adjustable power range, with a maximum power of less than 1mW. The LEDs and the PDs are arranged to create six source–detector separations, ranging from 10 mm to 60 mm. Furthermore, the probe has additional space for the placement of an ultrasound probe during simultaneous NIRS/ultrasound imaging. 

The Multimodal sensor has a sampling rate of 20 Hz. Testing on penetration depth was performed on phantoms mimicking the maternal tissues and the placenta, following the procedure previously described in [12], which indicated that the sensor is sensitive to signal change at a depth of 25 mm [12]. In addition, the tissue oxygenation measurement using the NIRS sensors was tested against a commercial time–domain NIRS system (TRS-41 system, Hamamatsu photonics, Japan), which resulted in an average of 2.7 ± 1.8% difference between the sensor and the commercial system [12].

### 2.2. Participants

The study protocols for both sensors were approved by the Wayne State University Human Investigations Committee Institutional Review Board (090717MP4E, approved on 9/27/2017). The NIRS sensor was utilized on patients seen at the Detroit Medical Center (DMC)/Wayne State University/the Perinatology Research Branch (Detroit, MI, USA) in March 2020 to measure placental oxygenation levels in 12 pregnant women (age: 27.1 ± 4.2 years) in their third trimester. The Multimodal sensor was also used in patients at the DMC to perform physiological signal measurements in 24 pregnant women (age: 28.3 ± 5.7 years) after 26 weeks of pregnancy in September 2022. The patients were recruited from various clinics, including the High Risk Pregnancy Unit, Obstetric Ultrasound Unit, Antenatal Care Clinic, Labor and Delivery, and the Obstetric Triage. The demographic and characteristics of the participants who were measured with the NIRS sensor and the Multimodal sensor are listed in Supplementary Table S1 and Table S2, respectively. At the time of delivery, placental tissues were obtained from 32 of the 36 participants, who delivered at the DMC, for pathological study. The placental tissues were examined for issues following the Amsterdam classification system. 

### 2.3. Experimental Procedure

Only participants with an anterior or a lateral placenta were enrolled in this study. The enrollment process involved the participants signing a written consent form, physicians reviewing their medical records, and a clinical determination so that the study would not negatively impact the established inpatient monitoring process and, thus, the participants’ physical or mental health. The initial stage of the experiment involved locating and marking the position of the placenta on the abdominal surface, as well as measuring the thickness of the tissues above the placenta, using an ultrasound machine available at a clinic. Depending on the placenta’s location, a participant had one, two, or three measurement locations marked. The sensor was placed on each marked location for approximately five minutes during the experiment (Figure 1b). Upon the completion of data collection or in the presence of pain during labor, the experiment was terminated. 

### 2.4. Data Acquisition and Processing

After the placement of a sensor on the participant’s abdominal surface, the sensor was turned on and connected to a computer through a universal serial bus (USB) cable (the NIRS sensor) or Bluetooth (the Multimodal sensor). The data acquired from the sensors were displayed on a computer screen through a customized MATLAB-based (the NIRS sensor) or web-based (the Multimodal sensor) software. The light intensities emitting from the LEDs were adjusted through the software to obtain a good signal-to-noise ratio. Once the data quality was ensured, the data were recorded through the software and saved to the computer.

The data processing was performed in MATLAB (Version R2023a, MathWorks). The transabdominal placental oxygenation levels were calculated using the spatially-resolved spectroscopy method that was previously described [12], utilizing backscattered light measured from different source–detector pairs on the sensors. Because the Multimodal sensor has a high data acquisition rate (20 Hz), its data contain the information of maternal physiological signals. These signals, including heart rate and breathing rate, were derived from backscattered light, which was measured at a given wavelength from a given source–detector pair of an NIRS channel in the Multimodal sensor. Signals from any wavelength of any source–detector pair could be used as they all contain information about maternal physiological signals. The obtained signals were processed using bandpass filters at 0.2–0.7 Hz for breathing rate derivation and 0.7–2 Hz for heart rate derivation. Subsequently, a peak detection algorithm in MATLAB was employed to automatically identify the minimum and maximum peaks, which were then manually checked to remove incorrectly detected peaks. Finally, the distance between maximum peaks, or the peak interval, was used to calculate the heart rate and the breathing rate. The motion sensor embedded in the Multimodal sensor provides information about the movement of the maternal abdominal surface in three dimensions, which was used to detect fetal movement and uterine contraction.

### 2.5. Statistical Analysis

The placental oxygenation levels calculated from each sensor were averaged throughout a measurement at a location per a participant. For the analysis on the placental oxygenation level, the data were first calculated separately for each sensor, and then data collected from each sensor were combined, which resulted in a total of 36 participants. The participants in this study were divided into 4 different groups, based on pregnancy complications. To assess the difference in the placental oxygenation levels in these groups, single-factor, one-way analysis of variance (ANOVA) analyses were conducted. A statistical test yielding a *p*-value of less than or equal to 0.05 was considered to be statistically significant. If the ANOVA test resulted in significance, subsequent multiple unpaired, two-tailed, post-hoc *t*-tests were performed on each pair of groups to identify the specific group differences.

## 3. Results and Discussions

### 3.1. Patient Characteristics

Among the 12 participants measured with the NIRS sensor, four had an uncomplicated pregnancy, five had maternal pre-existing conditions/complications, five had placental pathologies, and two delivered neonates with complications. Ten of the twelve participants delivered at the DMC, and their placentas were sent for histological examination. Five placentas had abnormalities, such as inflammatory lesions, maternal vascular mal-perfusion lesions, and Focal villous edema. Details on the patients’ characteristics and pregnancy outcomes can be found in Supplementary Table S1.

Of the 24 participants measured with the Multimodal sensor, 10 patients had pre-existing medical conditions, such as chronic hypertension, asthma, type II diabetes mellitus, renal failure with dialysis, and prolactinoma, and five had severe pre-eclampsia (Supplementary Table S2). Twenty-two of the twenty-four participants delivered at the DMC and had their placentas examined histologically. Seventeen of the placentas exhibited issues, such as acute and/or chronic inflammatory lesions, acute funisitis and vasculitis, placental infarction, and/or lesions consistent with maternal vascular malperfusion. Unfortunately, there were also negative outcomes during the postpartum period: one participant endured postpartum hemorrhage, and seven infants had neonatal complications, including prematurity, hypoplastic left heart syndrome, SGA, the tetralogy of Fallot, and cardiac anomaly (Supplementary Table S2). 

### 3.2. Transabdominal Placental Oxygen Saturation

**Figure 2:**
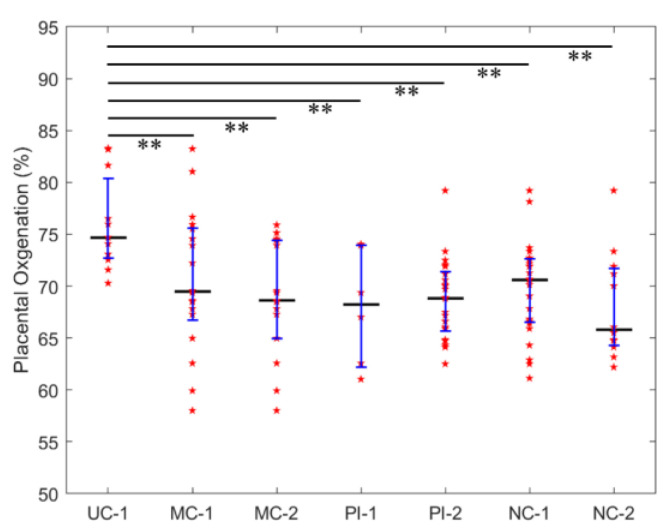
Transabdominal placental oxygenation from different groups. Oxygenation levels from different locations of all subjects are presented with red stars. Vertical line: median; error bar low: 1st quartile; error bar high: 3rd quartile. The number “1”, set at the end of the group label, corresponds to the data measured with the NIRS sensor, whereas “2” corresponds to the data measured with the Multimodal sensor. UC: uncomplicated pregnancy group; MC: maternal pre-existing conditions /complications group; PI: placental issues group; NC: neonatal complications group. Error bars represent standard deviation. ** Indicates a difference with statistical significance.

The transabdominal placental oxygenation levels measured with the NIRS sensor from 12 participants were divided into four groups: (1) the pregnancy without complications group (UC-1, n = four participants) showed placental oxygenation levels of 76.0 ± 4.4%; (2) the pregnancy with maternal pre-existing conditions/complications group (MC-1, n = six participants) showed transabdominal placental oxygenation levels of 70.5 ± 6.8%; (3) the pregnancy with placental pathologic abnormalities group (PI-1, n = five participants) showed placental oxygenation levels of 68.7 ± 5.6%; and (4) the pregnancy with neonatal complications group (NC-1, n = two participants) showed placental oxygenation levels of 68.0 ± 5.5% (Figure 2). 

The tissue oxygenation levels from 7 of the 24 participants, collected with the Multimodal sensor, were excluded from the analysis due to a large tissue thickness above the placenta (>25 mm). The data from the remaining 17 participants were divided into three groups: (1) the pregnancy with maternal pre-existing conditions/complications group (MC-2, n = nine participants) had transabdominal placental oxygenation levels of 68.7 ± 3.9%; (2) the pregnancy with placental pathologic abnormalities group (PI-2, n = eleven participants) had transabdominal placental oxygenation levels of 69.7 ± 4.5%; and (3) the pregnancy with neonatal complications group (NC-2, n = four participants) had transabdominal placental oxygenation levels of 68.0 ± 5.1% (Figure 2). 

The single factor ANOVA analysis of these seven groups of data that were acquired by both sensors reveals significant differences in the tissue oxygenation levels among the groups (*F*(6,103) = 3.6, *p* = 0.003). The post-hoc *t*-test analyses showed that in comparison to pregnancy without complications, pregnancy with maternal pre-existing conditions/complications, placental pathologic abnormalities, and/or neonatal complications had statistically lower transabdominal placental oxygenation levels (unpaired *t*-test, *p* < 0.001, Figure 2). 

Since both the sensors had similar transabdominal placental oxygenation level measurements (unpaired *t*-test, *p*-values > 0.05), the data were pooled together and were re-analyzed. The re-analysis confirmed similar results: pregnancy without complications had transabdominal placental oxygenation levels of 76.0 ± 4.4%; pregnancy with maternal pre-existing conditions/complications had transabdominal placental oxygenation levels of 69.5 ± 5.4%; pregnancy with placental pathologic abnormalities had transabdominal placental oxygenation levels of 69.4 ± 4.9%; and pregnancy with neonatal complications had transabdominal placental oxygenation levels of 68.0 ± 5.1%. A single factor ANOVA analysis revealed statistically significant differences among the groups (*F* (3,104) = 6.6, *p* = 0.0004). Post-hoc *t*-test analyses revealed that the pregnancy without complications group had significantly higher transabdominal placental oxygenation levels than the other three groups [*p* = 0.0005 (maternal pre-existing conditions/complications), *p* = 0.0001 (placental pathologic abnormalities), and *p* = 0.0001 (neonatal complications)].

### 3.3. Maternal Physiological Signals

Because the Multimodal sensor has a high sampling rate, it has the additional capabilities of measuring maternal heart rate and respiratory rate. Table 1 showed the maternal heart rate and respiratory rate of the 24 participants who were measured by the Multimodal sensor [“HR(Sensor)” and “BR (Sensor)”] at the time of assessment compared to maternal heart rate and respiratory rate extracted from the participants’ medical records [“HR (MR)” and “RR (MR)”]. From the participants’ medical records, the heart rate was available for all 24 of the participants, but the respiratory rate was missing for seven of the participants. The heart rate and the breathing rate showed widely fluctuating values during data acquisition. Nonetheless, 4 of the 24 participants had recorded heart rates greater than the range of heart rates measured by the Multimodal sensor (Table 1). Three of these four participants had recorded heart rates greater than 100 beats per minute (bpm). Moreover, 7 of the 17 participants had recorded respiratory rates lower than the range of respiratory rates obtained from the Multimodal sensor (Table 1).

### 3.4. Maternal Abdominal Surface Movements

**Figure 3:**
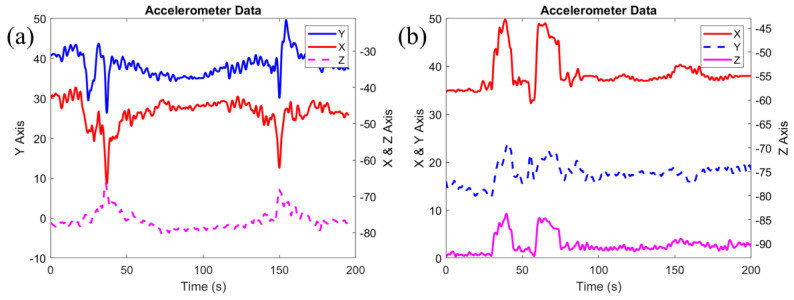
Movements in longitudinal (X), lateral (Y), and vertical (Z) directions of the motion sensor (**a**) measured during labor, with frequent contractions every two minutes; (**b**) measured when a mother reported multiple fetal movements.

Among the 24 participants who were measured with the Multimodal sensor, seven pregnant women were recruited from the Labor and Delivery unit. Two of the seven participants were in active labor, with frequent uterine contractions every two minutes (Participant 14) and every nine minutes (Participant 16). Figure 3a represents the data from the motion sensor acquired from Participant 14, who was having uterine contractions every two minutes. Within 3 min, the sensor had captured two contractions, which were approximately 2 min apart. During both of the contractions, movements in the x and y coordinates gradually decreased to minimum values at the beginning of a contraction, but eventually increased to the initial values before the contraction. However, movements in the z-coordinate increased during a contraction and decreased upon its completion. For all the coordinates, the first captured contraction caused a greater movement and for a longer duration than the second captured contraction. Hence, there is a potential to measure the strength and length of a contraction using the Multimodal sensor by calculating the maximum/minimum amplitude and the variation interval of the obtained signals. For Participant 16, one uterine contraction was detected with the motion sensor during data measurement.

During data acquisition with the Multimodal sensor, 6 of the 24 participants reported fetal movements (Participants 6, 8, 11, 12, 18 and 20). During most reported fetal movements, alterations in all the directions of the motion sensor were observed. Figure 3b displays an example of signals from the motion sensor when Participant 11 reported repeated fetal movements. For this participant, fetal movements resulted in increased signal values along the three coordinates. In general, variation in the longitudinal, lateral, and vertical directions of the motion sensor can be used to monitor fetal movements. 

### 3.5. Discussion

In this study, we demonstrated the efficiency of both sensors in measuring transabdominal placental oxygenation. We constantly observed a significantly lower transabdominal placental oxygenation level in pregnancies with maternal pre-existing conditions/complications, placental pathologic abnormalities, and/or neonatal complications than in those without complications. These findings confirm the close relationship between placental oxygenation and the presence of maternal and neonatal complications as well as placental pathologic abnormalities proposed in [12]. However, the results contrast with that of Wang et al., who were unable to find a difference in baseline placental oxygenation between women with normal and abnormal pregnancies, nor between normal and malperfused placentas [7]. This difference could be because of small sample sizes and the inclusion of various pregnancy complications in both studies. In addition to measuring placental oxygenation, we have demonstrated that the Multimodal sensor can measure maternal physiological signals, such as heart rate, respiratory rate, and uterine contraction, and fetal movements. Thus, the sensor can potentially assess maternal, placental, and fetal conditions concurrently to detect pregnancies that are at risk of poor outcomes and guide timely clinical interventions to improve pregnancy outcomes. Finally, the Multimodal sensor retains the features of being wearable, wireless, and convenient, opening avenues for non-invasive continuous monitoring and personalized care in the realm of obstetrics, marking a significant step toward improving maternal, fetal, and neonatal outcomes.

We found that maternal pre-existing conditions/complications during pregnancy, such as hypertension, severe pre-eclampsia, asthma, and type 2 diabetes mellitus, are important factors that significantly reduce maternal tissue oxygen saturation levels at the placenta. Previous studies reported conflicting information regarding the effect of pre-eclampsia on the placenta. Eskild et al. [13] reported that the increased blood pressure and endothelial permeability characteristics of pre-eclampsia might increase the blood flow to the intervillous space [14], resulting in an increase in oxygen for the placenta and fetus [15]. On the other hand, Cunningham et al. [16] and Tong et al. [17] suggested that the formation of occlusion due to trophoblast particles [18,19,20] and inappropriate, non-branching angiogenesis of fetal vessels [21] cause a reduction in uteroplacental blood flow in pre-eclampsia [16], which can result in hypoxia [16]. In addition, Matsuo et al. [22] reported that the arterio-venous oxygen saturation difference, a measure of placental oxygenation capacity, was lower for pre-eclamptic pregnancy with fetal growth restriction (FGR) compared to non-pre-eclamptic pregnancy with FGR. In addition to pre-eclampsia, previous studies have described the multiple negative effects of asthma on placental development and function. Uncontrolled asthma can reduce uterine blood flow and fetal oxygenation, leading to hypoxia, increased carbon dioxide levels in the blood, or acidosis [23]. Furthermore, the placental capillary volume also decreases, most notably in male fetuses, which may cause the dysfunction of the vascular endothelium and the smooth muscle of the placenta [23]. Hyper-ventilation can result in substantial fetal hypoxia and an increased fetal risk [24,25], such as the inhibition of terminal placental villous development and angiogenesis [26] and intrauterine growth restriction [27]. Moreover, altered placental blood flow resulting from maternal asthma was also inferred by viewing reduced vasodilation and vasoconstriction responses ex vivo after delivery [28]. Another maternal pre-existing condition that was reported to impact the placental oxygenation level is diabetes. Choo et al. [29] reported a lower birthweight/placental weight ratio, decreased placental efficiency, and various placental pathologic abnormalities, such as increased villous immaturity, fibrinoid necrosis, and increased villous vascularization [29], in diabetic patients [30,31,32,33,34]. In conclusion, these reports on the effects of pre-eclampsia, asthma, and diabetes on the placenta support our finding of significantly lower transabdominal placental oxygen saturation levels in pregnant women with pre-existing conditions/complications.

In addition to measuring transabdominal placental oxygenation, the Multimodal sensor was able to detect the maternal heart rate and breathing rate. However, in this study, we were not able to validate the accuracy of those measurements due to the lack of simultaneous monitoring with a medical-grade device. Instead, the heart rate and breathing rate were extracted from the patients’ medical records. However, most of the participants were either near term or in labor during data collection, which makes the comparison between our measurements and those within the medical record challenging. Previous studies have documented a gradual rise of the resting heart rate during pregnancy and a change in the heart rate in labor [35]. Musa et al. found an increase in the heart rate in the first stage of labor compared to the third trimester [36]. However, Robson et al. found an increase in cardiac output and arterial pressure due to an increase in stroke volume during the first stage of labor, but without an increase in the heart rate [37]. Similarly, the respiration rate was reported to vary throughout pregnancy and labor. Bossung et al. determined low breathing rates in gestational weeks 9 and 10, which increased around week 17 and then declined toward the end of pregnancy [38]. Therefore, to accurately assess the performance of the Multimodal sensor in monitoring the maternal heart rate and breathing rate, future studies should verify these measurements through continuous data acquisition during the simultaneous use of a medical-grade device and the updated sensor. 

The development of these wearable sensors does not only introduce a new avenue to monitor placental oxygenation continuously, but it also presents a great tool for remote monitoring. With the advantage of being wearable and wireless, the Multimodal sensor can be used both in the hospital or at home. We believe that the use of the sensor can potentially (1) help mitigate pregnancy complications; (2) reduce the time and economic burden for both patients and the healthcare system; and (3) provide a means for patients in low-resource areas to access pregnancy care. However, for the sensor to be used as a wearable, at-home monitoring device, the challenge of motion artifact needs to be addressed properly. In this study, even though the embedded motion sensor in the Multimodal sensor was sensitive to uterine contraction and fetal movement, the implications of the measured values from the sensor should be carefully evaluated. First, the measurements were performed when the mother was lying down, with minimum to no movement. However, to detect maternal contraction and/or fetal movement in daily activity, additional motion sensors will be required in future research to separate general maternal body movement from abdominal surface movement. Second, additional studies should be conducted to characterize the various types of movement that induce maternal abdominal surface movement through signal decoupling and the discrimination of acquired motion sensor data. 

The limitation of this pilot study is that it included a small patient population (n = 36 participants) and a small number of participants in a control group (n = 4 participants without complications). Among the 16 participants who had neither pre-existing conditions nor maternal complications, 12 of the participants presented with placental pathologic abnormalities or had neonates with complications. Given the variation in the patients’ background (race, ethical group, and general health), pregnancy conditions (maternal pre-existing conditions and complications), pregnancy outcomes (placental pathologic abnormalities, maternal and fetal adverse pregnancy outcomes), and gestational ages, it is uncertain whether the results found in this study can be applied for pregnant women in general. Therefore, future studies need to enroll a larger patient sample size, particularly including more pregnant women with no pre-existing conditions or maternal complications, to confirm the finding on the relationship between placental oxygenation levels and maternal pre-existing conditions, complications, or placental pathologic abnormalities. 

## 4. Conclusions

We have developed two sensors to continuously monitor placental oxygenation transabdominally: the NIRS (near-infrared spectroscopy) sensor, consisting of multiple NIRS channels, and the Multimodal sensor, which is an upgraded version of the NIRS sensor, with a higher data acquisition rate and an added motion sensor to monitor the maternal heart rate and respiration rate, fetal movements, and uterine contractions. Measurements with these sensors indicate that the transabdominal placental oxygenation level is negatively affected by maternal pre-existing conditions/complications, placental pathologic abnormalities, and/or neonatal complications. This result proves the potential of these sensors as remote, wearable devices for the detection of intrauterine hypoxia. Additionally, added features in the Multimodal sensor allow it to be able to detect other complications in both the mother and the fetus, which may assist in the treatment of diseases in pregnancy to prevent adverse outcomes.

## Figures and Tables

**Table 1:** Maternal heart rate (HR) and respiratory rate (RR) from the medical records (MR) and the Multimodal sensor. NA indicates values that were not available. Heart rate is provided in beats per minute (bpm) and respiratory rate is provided in breaths per minute. Vital signs from the medical record and the Multimodal sensor were not simultaneously measured.

Patient	HR (MR)	RR (MR)	HR (Sensor)	BR (Sensor)
1	94	18	80.0 ± 11.0	16.1 ± 1.2
2	73	16	72.8 ± 3.9	23.5 ± 1.9
3	69	18	69.4 ± 9.5	22.3 ± 4.3
4	94	18	90.0 ± 4.1	17.9 ± 1.3
5	92	22	88.5 ± 3.3	24.1 ± 2.0
6	86	16	83.4 ± 12.9	19.8 ± 3.0
7	88	17	76.2 ± 9.7	23.1 ± 3.5
8	85	NA	76.9 ± 9.9	20.2 ± 3.1
9	106	NA	93.7 ± 6.5	17.5 ± 0.7
10	85	20	78.8 ± 2.7	21.0 ± 1.4
11	76	16	72.4 ± 8.9	20.1 ± 2.2
12	86	NA	88.5 ± 7.6	27.3 ± 3.0
13	104	20	100.7 ± 13.0	30.5 ± 4.9
14	92	18	92.4 ± 5.9	26.6 ± 3.2
15	96	18	86.6 ± 10.2	18.8 ± 4.4
16	75	16	72.1 ± 5.9	20.4 ± 1.5
17	131	19	83.6 ± 7.0	16.1 ± 3.0
18	86	NA	87.0 ± 15.4	25.1 ± 5.5
19	68	18	66.3 ± 11.0	21.9 ± 2.1
20	78	NA	80.7 ± 19.8	23.7 ± 2.0
21	112	20	86.0 ± 15.8	20.2 ± 1.3
22	72	16	86.6 ± 14.8	20.8 ± 6.1
23	90	NA	80.3 ± 13.7	24.2 ± 3.3
24	108	NA	74.8 ± 9.2	24.1 ± 4.3

## Data Availability

The datasets used and/or analyzed during the current study are available from the corresponding author on reasonable request.

## Supplementary Materials

The following supporting information can be downloaded at: https://www.mdpi.com/article/10.3390/bios14100481/s1, Table S1: Pregnancy and outcome information of 12 participants measured with NIRS sensor. P: participant; GA: gestational age, NA: not available; AA: African American; WH: white. The “GA-1” column presents gestational age at measurement. The “GA-2” column presents gestational age at delivery. GA values are presented in clinical standard notation. For example, 33.4 indicates 33 weeks and 4 days of gestation. Table S2: Pregnancy and outcome information of 24 participants measured with Multimodal sensor V.2. P: participant; GA: gestational age, NA: not available; AA: African American; WH: white., AI: American Indian, BR: biracial; NICU: neonatal intensive care unit, SGA: small for gestational age. The “GA-1” column presents gestational age at measurement. The “GA-2” column presents gestational age at delivery. GA values are presented in clinical standard notation. For example, 33.4 indicates 33 weeks and 4 days of gestation.

## Abbreviations

functional Magnetic Resonance Imaging	fMRI
intrauterine growth restriction	IUGR
near-infrared spectroscopy	NIRS
cardiotocography	CTG
small-for-gestational-age	SGA
polydimethylsiloxane	PDMS
light-emitting diode	LED
photodetectors	PDs
Detroit Medical Center	DMC
analysis of variance	ANOVA
pregnancy without complications	UC
maternal pre-existing conditions/complications	MC
placental pathologic abnormalities	PI
pregnancy with neonatal complications	NC
heart rate	HR
respiratory rate	RR
universal serial bus	USB

## References

[1] Thompson L.P., Crimmins S., Telugu B.P., Turan S. (2015). Intrauterine hypoxia: Clinical consequences and therapeutic perspectives. Res. Rep. Neonatol..

[2] Hutter D., Jaeggi E. (2010). Causes and mechanisms of intrauterine hypoxia and its impact on the fetal cardiovascular system: A review. Int. J. Pediatr..

[3] Habek D., Habek J.C., Jugović D., Salihagić A. (2002). Intrauterine hypoxia and sudden infant death syndrome. Acta Medica Croat. Cas. Hravatske Akad. Med. Znan..

[4] Nordstrom L., Arulkumaran S. (1998). Intrapartum fetal hypoxia and biochemical markers: A review. Obstet. Gynecol. Surv..

[5] Abramowicz J., Sheiner E. (2008). Ultrasound of the placenta: A systematic approach. Part II: Functional assessment (Doppler). Placenta.

[6] Ražem K., Kocijan J., Podbregar M., Lučovnik M. (2020). Near-infrared spectroscopy of the placenta for monitoring fetal oxygenation during labour. PLoS ONE.

[7] Wang L., Cochran J.M., Ko T., Baker W.B., Abramson K., He L., Schwartz N. (2022). Non-invasive monitoring of blood oxygenation in human placentas via concurrent diffuse optical spectroscopy and ultrasound imaging. Nat. Biomed. Eng..

[8] Hasegawa J., Nakamura M., Matsuoka R., Mimura T., Ichizuka K., Sekizawa A., Okai T. (2010). Evaluation of placental function using near infrared spectroscopy during fetal growth restriction. J. Perinat. Med..

[9] Mah A.J., Nguyen T., Ghazi Zadeh L., Shadgan A., Khaksari K., Nourizadeh M., Zaidi A., Park S., Gandjbakhche A.H., Shadgan B. (2022). Optical Monitoring of breathing patterns and tissue oxygenation: A potential application in COVID-19 screening and monitoring. Sensors.

[10] Kulkarni M.B., Rajagopal S., Prieto-Simón B., Pogue B.W. (2024). Recent advances in smart wearable sensors for continuous human health monitoring. Talanta.

[11] Nguyen T., Park S., Park J., Sodager A., George T., Gandjbakhche A. (2024). Application of the Single Source—Detector Separation Algorithm in Wearable Neuroimaging Devices: A Step toward Miniaturized Biosensor for Hypoxia Detection. Bioengineering.

[12] Nguyen T., Khaksari K., Khare S.M., Park S., Anderson A.A., Bieda J., Gandjbakhche A.H. (2021). Non-invasive transabdominal measurement of placental oxygenation: A step toward continuous monitoring. Biomed. Opt. Express.

[13] Eskild A., Strøm-Roum E.M., Haavaldsen C. (2016). Does the biological response to fetal hypoxia involve angiogenesis, placental enlargement and preeclampsia?. Paediatr. Perinat. Epidemiol..

[14] Stosur S. (2011). Serum water analysis in normal pregnancy and preeclampsia. Am. Soc. Clin. Lab. Sci..

[15] Mayhew T.M. (2014). Estimating oxygen diffusive conductances of gas-exchange systems: A stereological approach illustrated with the human placenta. Ann. Anat. -Anat. Anz..

[16] Cunningham M.W., LaMarca B. (2018). Risk of cardiovascular disease, end-stage renal disease, and stroke in postpartum women and their fetuses after a hypertensive pregnancy, American Journal of Physiology-Regulatory. Integr. Comp. Physiol..

[17] Tong W., Giussani D.A. (2019). Preeclampsia link to gestational hypoxia. J. Dev. Orig. Health Dis..

[18] Burton G.J., Jones C.J. (2009). Syncytial knots, sprouts, apoptosis, and trophoblast deportation from the human placenta. Taiwan. J. Obstet. Gynecol..

[19] Roberts J.M., Escudero C. (2012). The placenta in preeclampsia. Pregnancy Hypertens. Int. J. Women’s Cardiovasc. Health.

[20] Toal M., Chan C., Fallah S., Alkazaleh F., Chaddha V., Windrim R.C., Kingdom J.C. (2007). Usefulness of a placental profile in high-risk pregnancies. Am. J. Obstet. Gynecol..

[21] Baergen R.N. (2011). Manual of Pathology of the Human Placenta.

[22] Matsuo K., Malinow A.M., Harman C.R., Baschat A.A. (2009). Decreased placental oxygenation capacity in pre-eclampsia: Clinical application of a novel index of placental function preformed at the time of delivery. J. Perinat. Med..

[23] Meakin A., Saif Z., Jones A., Aviles P.V., Clifton V. (2017). Placental adaptations to the presence of maternal asthma during pregnancy. Placenta.

[24] Giles W., Murphy V. (2013). Asthma in pregnancy: A review. Obstet. Med..

[25] Coleman M.T., Rund D.A. (1997). Nonobstetric conditions causing hypoxia during pregnancy: Asthma and epilepsy. Am. J. Obstet. Gynecol..

[26] Khaliq A., Dunk C., Jiang J., Shams M., Li X.F., Acevedo C., Ahmed A. (1999). Hypoxia down-regulates placenta growth factor, whereas fetal growth restriction up-regulates placenta growth factor expression: Molecular evidence for “placental hyperoxia” in intrauterine growth restriction. Lab. Investig. A J. Tech. Methods Pathol..

[27] Murphy V., Gibson P., Smith R., Clifton V. (2005). Asthma during pregnancy: Mechanisms and treatment implications. Eur. Respir. J..

[28] Clifton V.L., Giles W.B., Smith R., Bisits A.T., Hempenstall P.A., Kessell C.G., Gibson P.G. (2001). Alterations of placental vascular function in asthmatic pregnancies. Am. J. Respir. Crit. Care Med..

[29] Choo S., de Vrijer B., Regnault T.R., Brown H.K., Stitt L., Richardson B.S. (2023). The impact of maternal diabetes on birth to placental weight ratio and umbilical cord oxygen values with implications for fetal-placental development. Placenta.

[30] Lao T., Lee C.-P., Wong W.-M. (1997). Placental weight to birthweight ratio is increased in mild gestational glucose intolerance. Placenta.

[31] Strøm-Roum E.M., Haavaldsen C., Tanbo T.G., Eskild T.A. (2013). Placental weight relative to birthweight in pregnancies with maternal diabetes mellitus. Acta Obstet. Et Gynecol. Scand..

[32] Vambergue A., Fajardy I. (2011). Consequences of gestational and pregestational diabetes on placental function and birth weight. World J. Diabetes.

[33] Jirkovská M., Kučera T., Kaláb J., Jadrníček M., Niedobová V., Janáček J., Krejčí V. (2012). The branching pattern of villous capillaries and structural changes of placental terminal villi in type 1 diabetes mellitus. Placenta.

[34] Huynh J., Dawson D., Roberts D., Bentley-Lewis R. (2015). A systematic review of placental pathology in maternal diabetes mellitus. Placenta.

[35] Soehnchen N., Melzer K., de Tejada B.M., Jastrow-Meyer N., Othenin-Girard V., Irion O., Kayser B. (2011). Maternal heart rate changes during labour. Eur. J. Obstet. Gynecol. Reprod. Biol..

[36] Musa S.M., Adam I., Hassan N.G., Rayis D.A., Lutfi M.F. (2017). Maternal heart rate variability during the first stage of labor. Front. Physiol..

[37] Robson S., Dunlop W., Boys R., Hunter S. (1987). Cardiac output during labour. Br. Med. J. (Clin. Res. Ed.).

[38] Bossung V., Singer A., Ratz T., Rothenbühler M., Leeners B., Kimmich N. (2023). Changes in Heart Rate, Heart Rate Variability, Breathing Rate, and Skin Temperature throughout Pregnancy and the Impact of Emotions—A Longitudinal Evaluation Using a Sensor Bracelet. Sensors.

