# Supplementary material for: A Wireless and Wearable Multimodal Sensor to Non-Invasively Monitor Transabdominal Placental Oxygen Saturation and Maternal Physiological Signals
Source: Biosensors (Basel). 2024 Oct 7;14(10):481. doi: 10.3390/bios14100481 (PMC11506160; doi:10.3390/bios14100481)
Supplement: Supplementary file 1 [file biosensors-14-00481-s001.zip › biosensors-3216265-supplementary.pdf]

Article

A Wireless and Wearable Multimodal Sensor to Non-invasively Monitor Transabdominal Placental Oxygen Saturation and Maternal Physiological Signals

Thien Nguyen [1], Soongho Park [1,2], Asma Sodager [1], Jinho Park [1], Dahiana M. Gallo [3,4], Guoyang Luo [5,6], Roberto Romero [7,8,9] and Amir Gandjbakhche [1,*]

1. *Eunice Kennedy Shriver* National Institute of Child Health and Human Development, National Institutes of Health, 49 Convent Drive, Bethesda, MD 20892, USA; thien.nguyen4@nih.gov (T.N.); soongho.park@nih.gov (S.P.); jinho.park@nih.gov (J.P.)
2. Laboratory of Vascular Thrombosis and Inflammation, National Heart, Lung, and Blood Institute, National Institutes of Health, 49 Convent Dr., Bethesda, MD 20814, USA
3. Department of Obstetrics and Gynecology, Wayne State University School of Medicine, 3990 John R. Street, Detroit, MI 48201, USA; dahiana.gallo@sluhn.org
4. Division of Maternal-Fetal Medicine, Department of Obstetrics & Gynecology, St. Luke's University Health Network, 701 Ostrum Street, Suite 303, Bethlehem, PA 18015, USA
5. Obstetrics & Gynecology at the University of Virginia School of Medicine, 1340 Jefferson Park Ave, Charlottesville, VA 22903, USA; guoyang.luo@inova.org
6. Department of Obstetrics & Gynecology, Division of Maternal Fetal Medicine, Fairfax Hospital, 3300 Gallows Rd, Falls Church, VA 22042, USA
7. Pregnancy Research Branch, Division of Obstetrics and Maternal-Fetal Medicine, Division of Intramural Research, *Eunice Kennedy Shriver* National Institute of Child Health and Human Development, National Institutes of Health, U.S. Department of Health and Human Services, Bethesda, MD 20892, USA; romeror@mail.nih.gov
8. Department of Obstetrics and Gynecology, University of Michigan, Ann Arbor, MI 48109, USA
9. Department of Epidemiology and Biostatistics, Michigan State University, East Lansing, MI 48824, USA

* Correspondence: gandjbaa@mail.nih.gov

Supplementary data:

Table S1: Pregnancy and outcome information of 12 participants measured with NIRS sensor. P: par-ticipant; GA: gestational age, NA: not available; AA: African American; WH: white. The "GA-1" column presents gestational age at measurement. The "GA-2" column presents gestational age at delivery. GA values are presented in clinical standard notation. For example, 33.4 indicates 33 weeks and 4 days of gestation.

P	GA -1	GA-2	Race	Preexisting Conditions	Maternal Complications	Neonatal Complications	Placental Pathology
**1**	33.4	39.1	AA	None	None	None	None
**2**	30.6	38.6	AA	None	None	None	Chronic inflammatory lesions
**3**	36.6	39.2	WH	None	Gestational hypertension	None	Acute inflammatory lesions
**4**	28.5	40.6	AA	None	None	Temperature instability	None
**5**	29.1	39.5	WH	None	None	None	None
**6**	38.6	39.2	AA	None	None	None	None

**7**	39.1	40.1	AA	None	Polyhydramnios	None	Maternal vascular mal-perfusion le-sions
**8**	31.3	38.5	AA	None	None	None	None
**9**	29.3	39.2	AA	Chronic hy-pertension	None	None	Acute inflamma-tory lesions
**10**	34.2	37.0	AA	Chronic hy-pertension	None	Mild intermittent tachypnea	Focal villous edema
**11**	33.1	40.4	AA	Asthma	None	None	NA
**12**	33.5	NA	AA	None	Short cervix	NA	NA

Table S2: Pregnancy and outcome information of 24 participants measured with Multimodal sensor V.2. P: participant; GA: gestational age, NA: not available; AA: African American; WH: white., AI: American Indian, BR: biracial; NICU: neonatal intensive care unit, SGA: small for gestational age. The "GA-1" column presents gestational age at measurement. The "GA-2" column presents gesta-tional age at delivery. GA values are presented in clinical standard notation. For example, 33.4 in-dicates 33 weeks and 4 days of gestation.

P	**GA-1**	**GA-2**	**Race**	**Preexisting Condition**	**Maternal Compli-cations**	**Neonatal Compli-cations**	**Placental Pathology**
1	41.0	41.0	AA	None	None	None	Acute inflammatory lesions
2	32.2	34.0	AA	None	None	Preterm birth, hy-perbilirubinemia	Chorionic villous edema, patchy
3	41.2	41.3	AA	Asthma	Preeclampsia with severe features	None	None
4	35.6	39.1	> 1 race	None	None	None	Acute inflammatory lesions
5	26.0	30.0	AA	None	None	Prematurity	Acute inflammatory lesions
6	35.0	41.1	AI	Left hydro-nephrosis	None	None	Sub-amniotic hemor-rhage Intraplacental throm-bus
7	38.4	38.4	Asia n	None	None	NICU admission/ hypoplastic left heart syndrome	Placental infarct
8	34.4	40.5	AA	None	Postpartum hemor-rhage	None	Acute inflammatory lesions
9	40.3	41.0	AA	None	None	None	Small intervillous thrombus
10	34.3	34.3	WH	Gestational hy-pertension	Severe preeclamp-sia	Prematurity	Maternal vascular mal-perfusion lesions
11	33.5	37.2	AA	Prolactinoma	None	SGA	None
12	36.0	39.2	AA	None	None	None	Small intervillous thrombus

13	34.4	40.0	AA	Hereditary spastic paraplegia, asthma, chronic hypertension	Superimposed severe preeclampsia	None	Acute inflammatory lesions
14	39.1	39.1	AA	Asthma	None	None	None
15	31.2	38.0	BR	None	Severe preeclampsia, group A strep bacteremia, pulmonary edema	No report	NA
16	38.6	38.6	Asian	Type 2 diabetes mellitus	None	Tetralogy of Fallot	Chronic inflammatory lesions
17	32.3	37.0	AA	Renal failure with dialysis	None	None	Chronic villitis of unknown etiology
18	39.1	41.1	BR	None	None	None	Acute inflammatory lesions
19	38.6	38.6	AA	None	Preeclampsia with severe features	None	Acute inflammatory lesions
20	35.2	37.1	BR	None	None	SGA	None
21	33.0	37.1	AA	Type 2 diabetes, chronic hypertension	None	No report	NA
22	34.6	39.0	AA	None	None	None	Acute inflammatory lesions
23	34.3	38.4	AA	None	None	None	Acute inflammatory lesions
24	31.6	34.1	AA	Asthma	None	Preterm birth	None
